# Roles of transmembrane protein 135 in mitochondrial and peroxisomal functions - implications for age-related retinal disease

**DOI:** 10.3389/fopht.2024.1355379

**Published:** 2024-01-31

**Authors:** Michael Landowski, Purnima Gogoi, Sakae Ikeda, Akihiro Ikeda

**Affiliations:** ^1^Department of Medical Genetics, University of Wisconsin-Madison, Madison, WI, United States; ^2^McPherson Eye Research Institute, University of Wisconsin-Madison, Madison, WI, United States

**Keywords:** TMEM135, AMD, mitochondria, peroxisomes, lipid, DHA, RPE, photoreceptors

## Abstract

Aging is the most significant risk factor for age-related diseases in general, which is true for age-related diseases in the eye including age-related macular degeneration (AMD). Therefore, in order to identify potential therapeutic targets for these diseases, it is crucial to understand the normal aging process and how its mis-regulation could cause age-related diseases at the molecular level. Recently, abnormal lipid metabolism has emerged as one major aspect of age-related symptoms in the retina. Animal models provide excellent means to identify and study factors that regulate lipid metabolism in relation to age-related symptoms. Central to this review is the role of transmembrane protein 135 (TMEM135) in the retina. TMEM135 was identified through the characterization of a mutant mouse strain exhibiting accelerated retinal aging and positional cloning of the responsible mutation within the gene, indicating the crucial role of TMEM135 in regulating the normal aging process in the retina. Over the past decade, the molecular functions of TMEM135 have been explored in various models and tissues, providing insights into the regulation of metabolism, particularly lipid metabolism, through its action in multiple organelles. Studies indicated that TMEM135 is a significant regulator of peroxisomes, mitochondria, and their interaction. Here, we provide an overview of the molecular functions of TMEM135 which is crucial for regulating mitochondria, peroxisomes, and lipids. The review also discusses the age-dependent phenotypes in mice with TMEM135 perturbations, emphasizing the importance of a balanced TMEM135 function for the health of the retina and other tissues including the heart, liver, and adipose tissue. Finally, we explore the potential roles of TMEM135 in human age-related retinal diseases, connecting its functions to the pathobiology of AMD.

## 1 Introduction

The retina is particularly sensitive to the effects of aging (1), allowing researchers an *in vivo* system to discover critical genes and pathways important in mitigating the aging process using model organisms. As wild-type mice age, it is common to observe decreases in visual function (2, 3), presence of sub-retinal pigmented epithelium (RPE) deposits (4), appearances of RPE multinucleation (5), formation of cataracts (6), development of ectopic synapses (7) and signs of neuroinflammation (7, 8) in their retinas. However, the pathways responsible for the generation of these age-dependent retinal pathologies is unknown.

Mouse genetic methodologies have been instrumental to our understanding of the molecular underpinnings of the retinal aging process. A noteworthy example of harnessing mouse forward genetics to identify a critical gene involved in retinal aging comes from the study of the *FUN025* mice. The *FUN025* mice originated from a *N*-ethyl-*N*-nitrosourea (ENU) mutagenesis screen and were identified as a strain showing progressive age-dependent retinal pathologies including photoreceptor cell degeneration, ectopic synapse formation, and increased retinal stress with an earlier onset (as early as two months of age) and faster rate compared to wild-type C57BL/6J mice (9–11). The progressive retinal pathologies in *FUN025* mice differ from rapid retinal degeneration observed in mice with mutations in phosphodiesterase 6B (*Pde6b*) (12), rhodopsin (*Rho*) (13), and other genes linked with inherited retinal diseases in humans that is complete by two to three weeks of age (14). Importantly, the unique spatial pattern of progression for age-dependent retinal pathologies in wild-type C57BL/6J mice from the peripheral to the central retina (7) is maintained in the retina of *FUN025* mice albeit with earlier onset and faster progression (11), indicating that the retinal aging process is accelerated in *FUN025* mice.

Identification of the gene responsible for the age-dependent retinal phenotypes in the *FUN025* strain can lead to new insights into pathways contributing to retinal aging. Positional cloning of the *FUN025* line revealed a point mutation in the donor splice site of exon 12 of the transmembrane protein 135 (*Tmem135*) gene (11). Overexpression of *Tmem135* can prolong longevity in nematodes when exposed to cold temperatures (15) but no previous studies have correlated *Tmem135* with aging in mammals. The discovery of *Tmem135* as a gene implicated in retinal aging of mammals lead to subsequent studies on the function of *Tmem135* in cells and mice as well as associations between the pathways affected by *Tmem135* and age-related retinal diseases such as age-related macular degeneration (AMD). Intriguing similarities have been observed between retinal abnormalities in *Tmem135^FUN025^* mutant mice and retinal pathologies in AMD patients (11), as well as between gene expression profiles of the *Tmem135^FUN025^* mutant eyecups and RPE/choroid samples from multiple stages of AMD (16).

In this review, we summarize the current literature on *Tmem135*. We provide an overview of the molecular functions of TMEM135 that are critical for the regulation of mitochondria, peroxisomes, and lipids. We also describe the age-dependent phenotypes of mice with perturbations in *Tmem135*, highlighting the concept that proper balance of *Tmem135* function is vital for the health of the retina and other tissues such as the heart, liver, and adipose tissue. Lastly, we postulate about the possible roles of TMEM135 in human age-related retinal diseases by relating the roles of TMEM135 to the pathobiology of AMD.

## 2 Molecular functions and roles of TMEM135

*Tmem135*, also known as peroxisomal membrane protein 52 (*Pmp52*), encodes a 52 kilodalton protein with five transmembrane domains that is highly conserved across multiple species (15). Protein domains of TMEM135 also share similarities with members of the Tim17 protein family, which are central components of translocases of the mitochondrial inner membrane that are important for mitochondrial biogenesis (17). Based on this information, it is unsurprising that the TMEM135 protein shows colocalization with both mitochondria (11, 15) and peroxisomes (18–23). TMEM135 can be also found on lipid droplets, but this localization may be contingent on cellular stress such as microbial infections (24) and cold exposure (15). Further evidence indicates that TMEM135 translocates from peroxisomes to mitochondria (25), suggesting that TMEM135 is involved in functional interaction between mitochondria and peroxisomes (26). Here, we will summarize studies on the molecular roles of TMEM135, which suggest that TMEM135 is likely a multi-functional protein involved in the regulation of mitochondria and peroxisomes.

### 2.1 TMEM135 is a mitochondrial fission regulator

Earlier work indicates an important role of TMEM135 in “mitochondrial dynamics” (11), which is the collective term for biogenesis, fusion, fission, and mitophagy events required to preserve mitochondrial integrity within cells during times of cellular and nutritional stress (27). Fibroblasts from mice with the *Tmem135^FUN025^* mutation that causes the loss of TMEM135 function had overly elongated mitochondrial networks that manifested in decreased number and increased size of mitochondria (11), while fibroblasts with overexpression of wild-type *Tmem135* had fragmented mitochondria that were more abundant and smaller than wild-type controls (11). Colocalization between TMEM135 and a mitochondrial fission factor, dynamin-related protein 1 (DRP1), was observed at sites of mitochondrial fission (11), suggesting that TMEM135 may regulate mitochondrial fission through interaction with DRP1.

**Figure 1:**
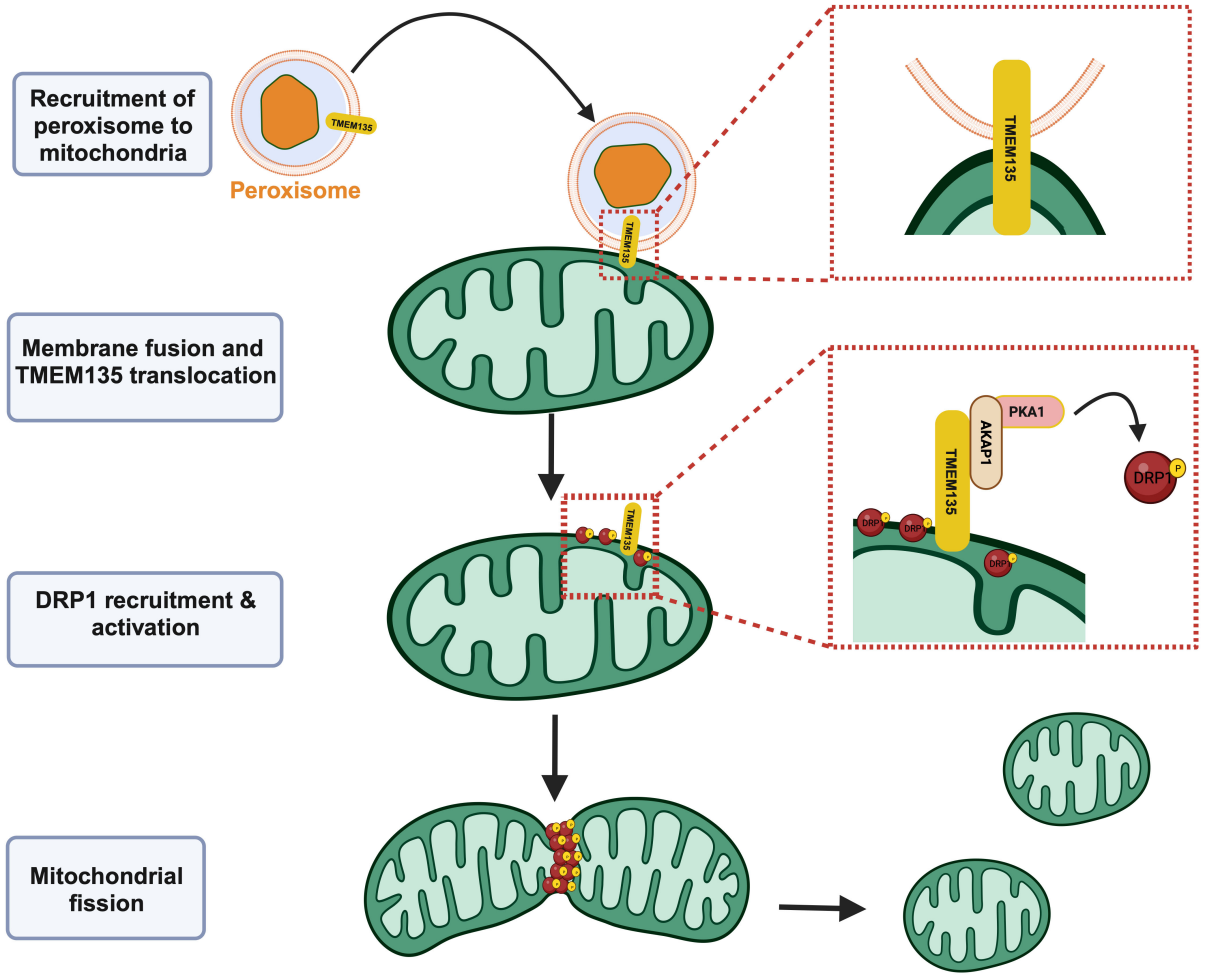
TMEM135 acts as a mitochondrial fission factor. Peroxisomes can interact with mitochondria, leading to the translocation of TMEM135 from the peroxisomal membrane to the mitochondrial outer membrane. The movement of TMEM135 from peroxisomes to mitochondria precipitates the recruitment of DRP1 and other proteins including AKAP1 and PKA1 that eventually leads to the activation of DRP1. Activated DRP1 causes fission of mitochondria. Image created using Biorender.com. TMEM135, transmembrane protein 135. DRP1, dynamin-related protein 1. AKAP, A-kinase anchor protein 1. PKA1, protein kinase A 1. [Figure adapted from Hu et al. (25)].

A recent study further elucidated the molecular function of TMEM135 as a regulator of DRP1, and thus, mitochondrial fission and its importance in the interaction between peroxisomes and mitochondria (25). Hu et al. observed that mitochondria appear overly fused in brown adipose tissue cultured from mice with the adipose tissue specific peroxisome deficiency, leading to impaired thermogenesis (25). Proteomic analysis of the mitochondria isolated from the peroxisome deficient brown adipocytes after cold exposure revealed TMEM135 as the most decreased protein (25), suggesting its involvement in the peroxisomal regulation of mitochondrial fission. The absence of TMEM135 on the mitochondria after cold exposure of the peroxisome deficient brown adipocytes indicated the prerequisite of TMEM135 to translocate from peroxisomes to mitochondrial membranes for the initiation of mitochondrial fission (**Figure 1**) (25). Investigation of the DRP1 phosphorylation state indicated that TMEM135 promotes protein kinase A (PKA)-dependent phosphorylation of DRP1 and its recruitment to mitochondria (25), defining the mechanism through which TMEM135 promotes mitochondrial fission. It was also shown that the translocation of TMEM135 from peroxisomes to mitochondria depends on plasmalogens (25), a class of glycerophospholipids containing a vinyl-ether and ester bond that are dependent on peroxisomes for their production (28). These findings add to the growing substantiation of an intimate relationship between peroxisomes and mitochondria that is needed for proper mitochondrial dynamics and homeostasis (29–33).

### 2.2 TMEM135 is an exporter of DHA from peroxisomes

A role of TMEM135 in lipid homeostasis was first indicated by transcriptomic profiling of retinal tissues isolated from mice with the *Tmem135^FUN025^* mutation. The retinal phenotypes of *Tmem135^FUN025/FUN025^* mutant mice correlated with increased expression of genes involved in fatty acid metabolism, cholesterol metabolism, and steroid metabolic processes (16), suggesting that the function of TMEM135 is important for the regulation of lipid synthesis. In support of this notion, age-dependent progression of neutral lipid and cholesterol accumulation was observed in the eyecups of *Tmem135^FUN025/FUN025^* mutant mice (16).

The relationship between TMEM135 and lipid metabolism was further defined through a high-throughput and semi-quantitative lipidomics analysis of *Tmem135^FUN025/FUN025^* mutant tissues. Untargeted profiling of intact lipid species using liquid chromatography with tandem mass spectrometry (LC-MS/MS) in the livers, retinas, hearts, and plasmas of *Tmem135^FUN025/FUN025^* mutant mice showed that each tissue had robust decreases in lipids containing Docosahexaenoic acid (DHA or C22:6n3) compared to wild-type control mice (34). Since all lipid classes that are known to harbor DHA were affected by the *Tmem135^FUN025^* mutation, which was confirmed by gas chromatography mass spectrometry (GC-MS) (34), it became apparent that TMEM135 has a major task in cellular DHA homeostasis.

**Figure 2:**
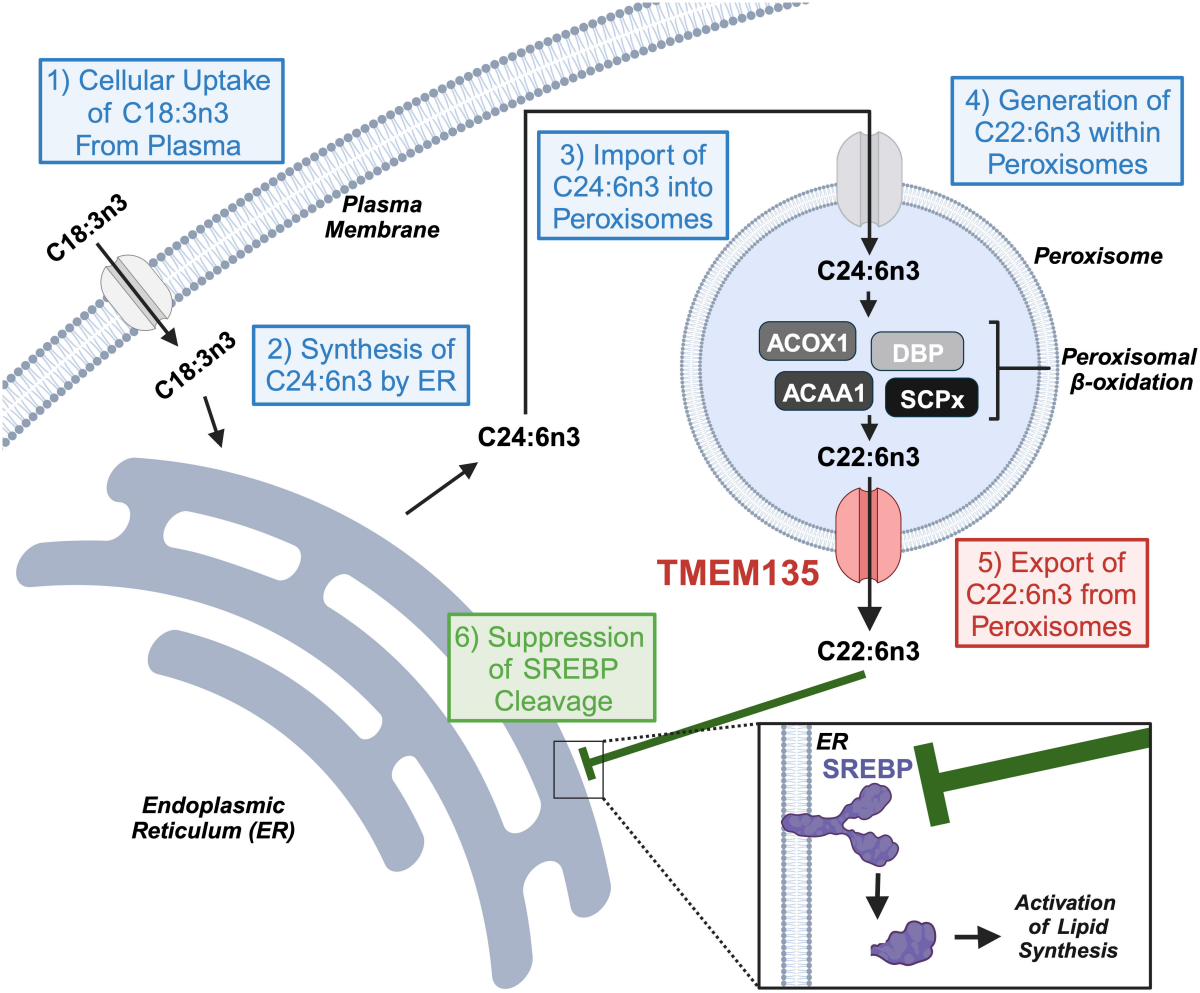
TMEM135 exports docosahexaenoic acid (DHA, C22:6) from peroxisomes to regulate cellular lipid synthesis. Cells utilize the ‘Sprecher pathway’ to synthesize DHA. (1) Cells uptake C18:3n3 from the blood and send it to the endoplasmic reticulum (ER). (2) The ER transforms C18:3n3 to C24:6n3 through a series of elongation and desaturation steps. (3) C24:6n3 migrates to the peroxisome for its import into its matrix. (4) Peroxisomal beta-oxidation enzymes (ACOX1, DBP, ACAA1, and SCPx) digest C24:6n3 to C22:6n3. (5) TMEM135 transports C22:6n3 from the peroxisomal matrix to the cytoplasm for its use by cells. (6) C22:6n3 accumulates within the ER to prevent the cleavage of the transcription factor SREBP and its activation of genes involved in lipid synthesis. Image created using Biorender.com. ACOX1, acyl-CoA oxidase 1. DBP, D-bifunctional protein. ACAA1, acetyl-coenzyme A acyltransferase 1. SCPx, sterol carrier protein x. SREBP, sterol response element binding protein.

DHA is an omega-3 polyunsaturated fatty acid (PUFA) important for neuronal development and function as well as an important mediator of inflammation and disease (35). The concentration of DHA within tissues results from the contribution of this omega-3 PUFA from dietary sources and endogenous production within cells (36). Since the diet consumed by *Tmem135^FUN025/FUN025^* mutant mice did not contain DHA (34), it must originate from the endogenous production from the ‘Sprecher pathway’ of DHA synthesis that takes place in the ER and completes in peroxisomes in these animals (37). The ER possesses desaturases [fatty acid desaturase 1 (*Fads1*) and 2 (*Fads2*)] and elongases [elongation of very long chain fatty acids-like 2 (*Elovl2*) and 5 (*Elovl5*)] needed for the desaturation and elongation of dietary essential fatty acid 18:3n3 to generate C24:6n3 (38). Then, C24:6n3 is imported into peroxisomes for retroconversion to C22:6n3 by their beta-oxidation enzymes (39). The ER and peroxisomal components of the ‘Sprecher pathway’ were evaluated in the livers of *Tmem135^FUN025/FUN025^* mutant mice to determine the molecular basis of the diminished DHA concentrations due to the *Tmem135^FUN025^* mutation (34). Remarkably, there were no decreases in any of the components, and rather there were increases in the peroxisomal beta-oxidation enzymes required to produce DHA (34) indicating that reduced DHA in *Tmem135^FUN025/FUN025^* mutant mice does not result from a defect in the ‘Sprecher pathway’ of DHA synthesis. The remaining step where TMEM135 may play a role in the generation of DHA within cells is the export of DHA from peroxisomes (**Figure 2**). While the exact molecular mechanism is unknown, it is thought that there is a protein on peroxisomes capable of exporting DHA from these organelles (40). The results of the lipid and pathway investigation of *Tmem135^FUN025/FUN025^* mutant mice strongly suggested that TMEM135 has a critical function in exporting DHA from peroxisomes to deliver DHA to the ER for esterification into lipids (**Figure 2**) (34). This is consistent with the postulated function of TMEM135 involving the transport of metabolites between organelles (18, 41).

### 2.3 TMEM135 influences peroxisome proliferation

Changes caused by *Tmem135* perturbations affect the number of peroxisomes, organelles that have chief responsibilities connected with cellular metabolism through its interactions with mitochondria, lipid droplets, lysosomes, and ER (42). In cultured fibroblasts with the *Tmem135^FUN025^* mutation, there was an increase of peroxisomes, while fibroblasts overexpressing *Tmem135* showed reductions in peroxisomal number (34). It is known that peroxisome proliferation is in part mediated by the actions of the peroxisome proliferator activated receptor (PPAR) family of transcription factors (43). Peroxisomes and their protein contents were decreased in the livers of *Tmem135^FUN025/FUN025^* mutant mice upon genetic ablation of PPAR alpha (*Ppara*) (34), indicating that activation of PPARa signaling is involved in increasing peroxisome proliferation in these mice. While it is unclear what drives the changes in PPAR signaling due to the changes in TMEM135 function, it is possible that impaired DHA export from peroxisomes results in the generation of peroxisome-derived metabolites that interact with PPARs such as ether phosphatidylethanolamines (EtherPEs) known to activate the PPAR signaling (44) which is increased in *Tmem135^FUN025/FUN025^* mutant mice (34). More work is required to discern the molecular mechanism underlying the peroxisome proliferation changes observed in *Tmem135* mutant and overexpressing cells, and its relationship with the TMEM135 molecular function.

### 2.4 TMEM135 is a mediator of intracellular cholesterol trafficking

TMEM135 has been implicated to have a role in the distribution of intracellular cholesterol by two different studies (45, 46). First, *Tmem135* was identified in a shRNA screen for genes involved in trafficking of cholesterol from low-density lipoprotein (LDL) to the plasma membrane of HeLa cells (45). TMEM135 was further validated as a protein involved in intracellular cholesterol trafficking by knocking it down in HeLa cells, which resulted in fewer contacts between lysosomes and peroxisomes as well as decreased cholesterol in the plasma membrane (45). These results suggested that lysosome-peroxisome trafficking of cholesterol mediated by contacts between these organelles is impaired in *Tmem135* knockdown cells. This result was further confirmed in another study using RPE1 cells, an immortalized RPE cell line often utilized in cilia-focused research (46). The authors observed fewer lysosome-peroxisome contacts (46) and an increased accumulation of cholesterol in the lysosomes of cells with decreased *Tmem135* expression (18). After treatment with LDL, the knockdown of *Tmem135* expression impaired the ability of cholesterol from LDL particles to reach the ER. Accumulation of cholesterol was observed in the eyecups of *Tmem135^FUN025/FUN025^* mutant mice (16), which may occur due to defective cholesterol transport in these mice. Interestingly, accumulation of cholesterol in *Tmem135^FUN025/FUN025^* mutant eyecups coincided with upregulation of sterol regulatory element binding transcription factor 2 (SREBP2)-targeted genes that are involved in cholesterol metabolism including hydroxymethylglutaryl-CoA synthase (*Hmgcs1*), sterol O-acyltransferase 1 (*Soat1*), ATP-binding cassette subfamily A member 1 (*Abca1*), and ATP-binding cassette subfamily A member 1 (*Abcg1*) (16). It is worth exploring whether these biochemical and expression manifestations result from defective lysosome-peroxisome interactions in *Tmem135^FUN025/FUN025^* mutant mice. Moreover, based on the TMEM135 function in DHA export (34), it would be interesting to investigate whether DHA-esterified lipids influence membrane fluidity and interactions of these organelles.

## 3 Tissue-specific roles of TMEM135 and its relevance to human disease

**Figure 3:**
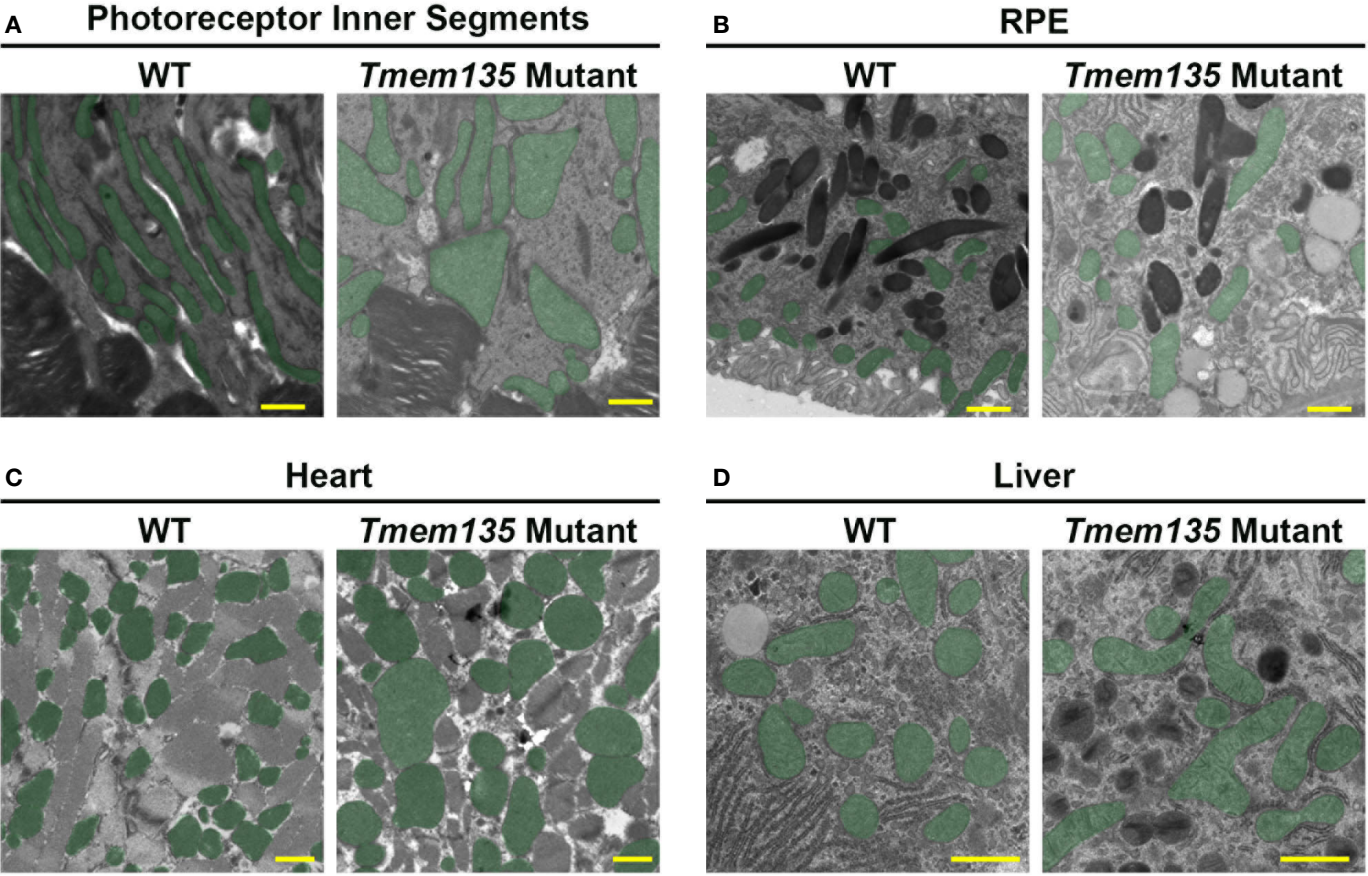
Mitochondrial shape in *Tmem135* mutant tissues. Representative electron micrographs of mitochondria in the photoreceptor inner segments **(A)**, retinal pigmented epithelium (RPE) **(B)**, heart **(C)**, and liver **(D)** of wild-type C57BL/6J (WT) and *Tmem135* mutant mice. Mitochondria are labeled in green. Note the enlarged mitochondria in all *Tmem135* mutant tissues relative to WT tissues. The magnifications for the photoreceptor inner segment and RPE micrographs are 8800X, heart micrographs are 7100X, and liver micrographs are 11500X. The scale bar in all micrographs represents 1 micron.

Insight into the significance of TMEM135 on metabolic functions of tissues can be gleaned by the age-related phenotypes in mice with modifications of *Tmem135* function. Remarkably, all tissues from mice with a homozygous mutation in *Tmem135* (*Tmem135^FUN025/FUN025^* mutant) or overexpression of *Tmem135* (*Tmem135* TG) show opposing differences in their mitochondrial shape (**Figure 3**) and number of peroxisomes (11, 34, 47–49). However, there are specific tissues that are more sensitive to the *Tmem135* mutation or overexpression (47, 50). There were also tissue-specific lipid adaptations (34). These findings indicate there is a tissue-specific reliance for TMEM135 on sustaining homeostasis through aging. Here, we will summarize the phenotypes of the *Tmem135^FUN025/FUN025^* mutant and *Tmem135* TG mice (**Table 1**) and the potential relevance of TMEM135 in human diseases associated with those phenotypes.

**Table 1:** Summary of features associated with TMEM135 loss of function and overexpression.

	Organellar Changes		Tissue Phenotypes				
	Mitochondria (11, 25, 47–49)	Peroxisomes (25, 34)	Eye		Heart (48)	Liver (34)	Fat (25)^****^
			Neural retina (11, 16, 47)	RPE (11, 16, 47)			
TMEM135 Loss of function	Overly-fused mitochondria	Increased number	▪ Photoreceptor cell degeneration ▪ Abnormal ERG ▪ Ectopic synapse development ▪ Müller glia activation ▪ Immune cell infiltration into the subretinal space	▪ Autofluorescence ▪ Increased thickness ▪ Increased density ▪ Decreased ERG c-wave amplitudes ▪ Lipid accumulation	▪ No phenotype^**^	▪ No phenotype^*^ • Less severe non-alcoholic fatty liver disease, less hepatic lipid accumulation when combined with the leptin mutation^***^	▪ Cold intolerance ▪ Increased diet-induced obesity ▪ Glucose intolerance and insulin resistance ▪ Increased adiposity
TMEM135 Overexpression	Excess mitochondrial fragmentation	Decreased number	▪ No phenotype^*^	▪ Degeneration ▪ Migration ▪ Vacuolization ▪ Dysmorphia ▪ Thinning	▪ Increased fibrosis ▪ Hypertrophy ▪ Large vacuoles between myofibrils	▪ No phenotype^**^	▪ Increased cold tolerance ▪ Decreased diet-induced obesity ▪ Glucose tolerance and increased insulin sensitivity ▪ Decreased adiposity

^*^Data is from study on Tmem135 mutant or overexpressing mice under normal unstressed conditions.

^**^ Data is not shown from studies on Tmem135 mutant or overexpressing mice.

^***^ Phenotypic difference was observed in Tmem135 mutant mice that are homozygous for the leptin mutation compared to homozygous leptin mutant mice.

^****^ Data is from studies using adipose-specific Tmem135 conditional knockout mice.

### 3.1 Ocular phenotypes of Tmem135 mutant mice

*Tmem135^FUN025/FUN025^* mutant mice develop an age-dependent photoreceptor cell degeneration (11, 16), which coincided with visual loss, ectopic synapse development, and neuroinflammation consisting of Müller glia activation and immune cell infiltration into the subretinal space (11, 16). *Tmem135^FUN025/FUN025^* mutant mice also showed changes in the RPE such as autofluorescence, increased thickness, increased density, decreased electroretinogram c-wave amplitudes, and lipid accumulation (11, 16, 47) (**Table 1**).

It is possible that the retinal phenotypes of the *Tmem135^FUN025/FUN025^* mutant mice may be occurring due to their deficiency of DHA (34). DHA has an important role in membrane fluidity of rod photoreceptor outer segments that is required for phototransduction (51). There are reports of other mouse models with retinal DHA deficiency including elongation of very-long-chain fatty acids-like 2 (*Elovl2*) mutant (52), acyl-CoA synthetase 6 (*Ascl6*) knockout (53), major facilitator superfamily domain containing 2A (*Mfsd2a*) knockout (54, 55), and adiponectin receptor 1 (*Adipor1*) knockout mice (56, 57) that show similar retinal pathologies to those observed in *Tmem135^FUN025/FUN025^* mutant mice (11, 16, 47).

It is also possible that the retinal phenotypes of *Tmem135^FUN025/FUN025^* mutant mice result from enhancement of mitochondrial fusion triggered by the *Tmem135* mutation (11). Boosting mitochondrial fusion in the retinas of *Tmem135^FUN025/FUN025^* mutant mice may lead to increased nutrient intake and metabolic stress as detected in other tissues caused by excessive mitochondrial fusion (58, 59). Supporting this idea, a NMR-based metabolomics study revealed an accumulation of metabolites from glucose, amino acid and lipid metabolic pathways in primary-cultured *Tmem135^FUN025/FUN025^* mutant RPE cells compared to wild-type RPE cells (50). In addition, mice with retinal metabolic stress such as RPE-specific vascular endothelial growth factor A (*Vegfa*) or superoxide dismutase 2 (*Sod2*) have thicker RPE like the *Tmem135^FUN025/FUN025^* mutant mice (11, 47). Future studies will need to be undertaken to determine the roles of the reduced DHA and overly fused mitochondria in *Tmem135^FUN025/FUN025^* mutant mice in regard to the development of their retinal pathologies.

### 3.2 Ocular phenotypes of Tmem135 TG mice

In contrast to *Tmem135^FUN025/FUN025^* mutant mice*, Tmem135* TG mice exhibit progressive RPE degenerative phenotypes including migration, vacuolization, dysmorphia, and thinning (47). Additionally, *Tmem135* TG mice displayed thinner myelin sheaths of axons in the optic nerve (49). However, unlike *Tmem135^FUN025/FUN025^* mutant mice, there were no signs of photoreceptor cell dysfunction or degeneration in *Tmem135* TG mice at least until one year of age (47).

The retinal phenotypes of *Tmem135* TG mice may result from their excess mitochondrial fragmentation (11, 47) and/or decreased peroxisome proliferation (34). It is believed that mitochondrial fission is important for the removal of damaged mitochondrial membranes in order to maintain mitochondrial function (58, 60). However, excessive mitochondrial fragmentation initiated by *Tmem135* overexpression could cause mitochondrial dysfunction in the RPE and lead to degeneration of this cell type. Similar to *Tmem135* TG mice, mice with RPE-specific ablation of transcription factor A, mitochondrial (*Tfam*) or PPARG coactivator 1 alpha (*Pgc-1α*) show attenuated mitochondrial function and degenerated RPE (61, 62). It is plausible that mitochondrial fragmentation in *Tmem135* TG could stem from their decreased peroxisomal proliferation (34). Inhibition of proper peroxisome biogenesis by eliminating peroxisomal biogenesis factor 3 (*Pex3*) or peroxisomal biogenesis factor 5 (*Pex5*) expression promoted mitochondrial fragmentation in mouse embryonic fibroblasts (33). Recent work has shown that genetic ablation of the multifunctional protein 2 (*Mfp2*; also known as hydroxysteroid (17-beta) dehydrogenase 4 or *Hsd17b4*) gene encoding D-bifunctional protein (DBP), which is a critical enzyme required for peroxisomal beta-oxidation (34, 63), specifically in RPE cells can lead to RPE degenerative changes (64). Interestingly, expression of *Mfp2* is decreased in the eyecups of *Tmem135* TG mice (34). Dissecting out the roles for mitochondrial fragmentation and decreased peroxisomal proliferation will be critical to determine the contributions of these organelles to the RPE degeneration observed in *Tmem135* TG mice.

### 3.3 Potential connection between TMEM135 and AMD

Studies from cell culture and animal experiments signal a substantial role of TMEM135 in energy homeostasis in aging. To date, there has been no direct association between TMEM135 and age-related retinal diseases including AMD. However, there are multiple levels of similarities between *Tmem135* mouse models and AMD including ocular pathologies and molecular and cellular changes (11, 16). Here, we will discuss potential involvement of TMEM135 in AMD pathogenesis.

#### 3.3.1 Mitochondrial changes in AMD

Dysfunction of mitochondria is an important pathobiological event in AMD (65–67). In the retina, there are numerous mitochondria within the photoreceptor and RPE cells (68). Mitochondria provide these retinal cells a constant supply of energy required for facilitating phototransduction and sequestering of reactive oxygen species from photons of light and other oxidative stresses (69). It is well established that aging disrupts mitochondrial homeostasis, which may predispose the retina to AMD (70). In particular, the RPE is thought to be the first tissue affected by AMD (71). Surveys of the mitochondria in the RPE of AMD-afflicted eyes uncovered robust decreases in their number and size (72). The changes in mitochondrial shape and number in the RPE of AMD donor retinas correlated with decreased mitochondrial proteins (73), increased mitochondrial DNA damage (74–76), and increased mitochondrial oxidative stress (77). To evaluate the functional consequences of these changes, RPE from human AMD donor eyes were cultured and assessed using the Seahorse Extracellular Flux Analyzer (78). These RPE cultures displayed a reduction in their glycolytic function (78) that has been validated by another group using a different method (79). The accrual of this work suggests targeting mitochondria is a viable treatment strategy for AMD as proposed by many groups in the AMD research field (80–91). It is of note that elamipretide, which targets mitochondria, recently failed in a phase 2 trial of geographic atrophy (92). However, this may still be a viable therapeutic for intermediate AMD (93), suggesting that targeting mitochondria at earlier stages of disease development may be more efficacious to treat early/intermediate AMD.

The deviations of normal mitochondria in the RPE of donor retinas hint at possible disruptions of mitochondrial dynamics in AMD. The proteins involved in mitochondrial fusion, fission, and autophagy were quantified in RPE cultures from AMD and control RPE cultures (94). Interestingly, upon treatment with the mitochondrial uncoupler FCCP, there was a disease-specific response in the RPE cultures from the AMD donor eyes including an increase of mitochondrial fission factor (MFF) (94). MFF is a protein necessary for mitochondrial fission (95), and its amplified expression correlated with the mitochondrial fragmentation typically observed in the RPE from AMD donor eyes. Since TMEM135 is a mitochondrial fission factor (11, 25, 47, 49), it may be involved in mitochondrial fragmentation in the RPE of AMD patients as well. Future work on the origins of mitochondrial fragmentation in AMD-diagnosed retinas is essential since the pharmacological inhibition of mitochondrial fission is thought to be a therapeutic target for non-exudative AMD (96).

#### 3.3.2 Decreased DHA-containing lipids in AMD

The mammalian retina, notably the rod photoreceptor outer segments, contains the highest density of DHA than any other tissue in the body, which is important for membrane fluidity of the outer segments (97). As previously discussed, DHA can originate from dietary sources or endogenous synthesis through the ‘Sprecher pathway.’ Dietary intake of DHA has been related to decreased risk for AMD, but these findings have not been well replicated as commented in other excellent reviews (98–100). This could be due in part to the preferential uptake of DHA in different forms such as triglycerides, phosphatidylcholine, or lysophosphatidylcholine by the retina (101). Recent work suggested an important contribution of rod photoreceptor-derived DHA in AMD (102). They showed through LC-MS/MS and MALDI-molecular imaging that there was a decrease of DHA-containing phosphatidylcholines in the peripheral retinas of AMD patients (102).

Transcriptomic analysis of donor retinas also supports the claim that there is less DHA in AMD-afflicted eyes. Integrated microarray and RNA-Seq datasets (GSE29801 and GSE135092) of RPE/choroid samples from AMD patients (16, 103–105) showed increased sterol regulatory element binding transcription factor 1 (*SREBP1*), a transcription factor required for the synthesis of fatty acids and cholesterol, and its target genes fatty acid synthase (*FASN*), fatty acid desaturase 1 (*FADS1*), and *FADS2* (16). Recent work showed that decreased DHA enhances the transcription of SREBP1 target genes (106) (**Figure 2**), suggesting that reduced DHA could cause increased SREBP signaling in AMD. Intriguingly, reduced DHA as well as increased expression of *Srebp1* and its target genes were also observed in *Tmem135^FUN025/FUN025^* mutant eyecup samples (16). Common molecular and pathological features between *Tmem135^FUN025/FUN025^* mutant mice and AMD patients suggest that the role of TMEM135 in peroxisomal export of DHA within retinal cells may be important in mitigating dysregulated lipid synthesis in AMD.

#### 3.3.3 Altered cholesterol metabolism in AMD

Cholesterol metabolism in AMD has been well investigated because there are large accumulations of esterified cholesterol within drusen, the pathological hallmark of AMD (107), and strong associations of AMD risk with genes involved in cholesterol transport (108–114). Disruptions to normal cholesterol homeostasis in the retina is thought to contribute to the onset of drusen in the sub-RPE space of the human retina (115). An understanding into retinal cholesterol metabolism comes from inquiries on the pathobiological nature of the retinal phenotype of ATP-binding cassette, subfamily A, member 4 (*Abca4*) knockout mice, which lead to discoveries on a vital role of dysregulated cholesterol trafficking as an important pathobiological event in the development of AMD-like pathologies in this model (116). *Abca4* knockout mice are characterized by the accumulation of A2E, a major lipofuscin fluorophore, that coincides with delayed dark adaptation (117, 118). A2E can displace cholesterol from the plasma membrane of RPE cells, accumulate cholesterol within RPE cells, and impede the ability of cholesterol efflux from these cells (119). Furthermore, cholesterol accumulation can induce ceramide production in the RPE and allow for complement-mediated damage on the RPE plasma membrane in *Abca4* knockout mice (120). Comparably, mice with the loss of Niemann-Pick Type C disease (NPC) intracellular cholesterol transporter 1 (NPC1), that lose the ability to transfer cholesterol from lysosomes to the cell, have impaired visual function and lipofuscin aggregation at 2 months of age (121). Given that TMEM135 has been shown to play a role in the intracellular trafficking of cholesterol between lysosomes and peroxisomes (45, 46), this function of TMEM135 may be important in sustaining cholesterol metabolism within the retina and preventing the formation of esterified cholesterol-enriched drusen in the sub-RPE space.

### 3.4 Effects of TMEM135 modulation in other tissues

There have been published associations with TMEM135 and other human medical conditions such as osteoporosis (122–127), breast cancer (128, 129), prostate cancer (130, 131), melanoma (132, 133), non-small lung cancer (134), glioblastoma multiforme (134), non-alcoholic fatty liver disease (135), cognitive disorders (136), and metabolic disease (25). Significance of TMEM135 functions have been also indicated by phenotypes in other tissues due to *Tmem135* mutation and overexpression. While readers are encouraged to refer to individual studies for details, we will summarize the phenotypes in other mouse tissues caused by modulation of *Tmem135*.

Overexpression of *Tmem135* impacts the heart along with the RPE (48). The hearts of *Tmem135* TG mice on a mixed C57BL/6J and FVB/NJ background show hypertrophy with increased fibrosis (48). Ultrastructural abnormalities such as large vacuoles co-occupying the space between myofibrils with mitochondria were observed in *Tmem135* TG hearts at varying severities (48). Cardiac phenotypes of *Tmem135* TG mice most likely derive from their mitochondrial fragmentation. Other mouse models with heart-specific conditional ablation of mitochondrial fusion display cardiac phenotypes comprising dilated cardiomyopathy and cardiac hypertrophy (137, 138). It remains unknown if mitochondrial fragmentation in *Tmem135* TG originates from mitochondria or peroxisomes as TMEM135 can translocate from peroxisomes to mitochondria for fission events (11, 25, 34).

While livers of *Tmem135^FUN025/FUN025^* mutant mice appear and function normally regardless of the remarkable changes in their hepatic peroxisomes and lipidome (34), physiological significance of these cellular changes could be observed when the *Tmem135^FUN025^* mutation was combined with the leptin mutation (*Lep^ob^*), which causes metabolic disease with significant hepatic lipid adjustments and dependency on functional peroxisomes in mice (139–141). Both male and female mice that are homozygous for *Tmem135* and leptin mutations (*Tmem135^FUN025/FUN025^/Lep^ob/ob^*) had lower body, liver, and gonadal fat pad weights compared to their *Lep^ob/ob^* counterparts (34). The *Tmem135^FUN025^* mutation also decreased the amount of plasma cholesterol by impairing the secretion of very low-density lipoprotein (VLDL) and LDL (142). Modifications of the classic obesity and dyslipidemia phenotype in *Lep^ob/ob^* mice by the *Tmem135^FUN025^* mutation correlated with attenuation of their non-alcoholic fatty liver disease (NAFLD) phenotypes. There were less severe NAFLD pathologies and hepatic lipid accumulation in *Tmem135^FUN025/FUN025^/Lep^ob/ob^* mice compared to *Lep^ob/ob^* mice (34). Together, these phenotypic changes suggest that impairment of TMEM135 function affects molecular pathways involved in the pathogenesis of metabolic disease with dysregulated lipid metabolism, which may include activation of PPAR signaling and increased peroxisome proliferation (34).

Most recently, adipose-specific deletion of *Tmem135* was shown to result in impaired thermogenesis and increased diet-induced obesity and insulin resistance in mice, revealing significant roles of TMEM135 in the brown fat and energy homeostasis (25). Conversely, *Tmem135* overexpression increased thermogenesis and prevented diet-induced obesity and insulin resistance (25). This study revealed aforementioned function of TMEM135 in the regulation of mitochondrial fission and placed TMEM135 as a critical mediator of the peroxisomal regulation of mitochondrial fission and thermogenesis (25). Additionally, the authors identified a single nucleotide polymorphism (SNP) in the human *TMEM135* gene associated with increased body mass index (BMI) in a Hispanic population (25). Functional studies indicated that this specific SNP in *TMEM135* reduces the function of the protein and may promote the occurrence of human metabolic diseases (25).

## 4 Future perspectives of TMEM135 research

There has been a wealth of knowledge on TMEM135 from new publications over the last several years. Yet, there are still many unanswered questions regarding this fascinating protein that need to be answered and its role in aging.

TMEM135 is increasingly recognized for its involvement in lipid homeostasis. Studies have shown that TMEM135 expression is elevated in conditions with lipid accumulation (15). However, the specific molecular mechanisms regulating TMEM135 expression remain to be fully elucidated. This aspect presents a potential avenue for future research to understand the role of TMEM135 in lipid homeostasis.

As detailed by the descriptions of mice with the loss-of-function mutation and overexpression of *Tmem135* in this review, TMEM135 has significant roles in multiple tissues. Future studies on TMEM135 should utilize mouse genetic approaches to interrogate the role of this protein in a tissue-specific manner. These strategies could include Cre-Lox technologies or viral vectors that modulate TMEM135 expression in discrete cells. This would prevent off-target effects that may confound the interpretation of the role of TMEM135 in a particular cell-type since TMEM135 has important roles in multiple cells. Also, it is unclear the contributions of dysregulated mitochondrial dynamics, DHA concentrations, peroxisome proliferation, and intracellular trafficking on the phenotypes of the *Tmem135* mutant and overexpressing mice. These pathways should be targeted to determine their contribution towards the phenotypes of these mice.

Previous studies highlight the crucial role of TMEM135 in connecting cellular organelles, particularly peroxisomes and mitochondria, as well as peroxisomes and lysosomes. The growing understanding of organelle interactions has attracted significant scientific interest. A key area of research is the role of these organelle interactions in the retina, particularly how they contribute to normal aging and their potential alteration in age-related conditions such as AMD.

Lastly, while genetic and molecular biological studies on TMEM135 have progressed, exploring the protein biochemically could yield valuable insights. For instance, identifying the structure of TMEM135, its binding partners, and small molecules that regulate its activity could pave the way for new therapeutic approaches targeting TMEM135 in metabolic and age-related diseases.

## 5 Conclusion

Aging is a significant stressor for tissues, but the molecular nature of aging remains a mystery. By using forward genetics, important genes and pathways involved in aging can be determined. In this review, we summarized the findings on TMEM135, an important player involved in retinal aging of mice that was uncovered through forward genetics. This unbiased phenotypic investigation led to the discovery of a critical protein involved in the regulation of mitochondrial and peroxisomal functions, as well as lipid homeostasis. Disruptions of TMEM135 function have detrimental consequences to the murine retina, but other tissues including the heart, liver, and adipose tissue can also be impacted by changes in TMEM135. Importantly, both the loss-of-function mutation in *Tmem135* and overexpression of *Tmem135* caused pathology development in mice, indicating the balance of TMEM135 function is required for normal healthy aging. Although no direct connection has been made between TMEM135 and age-related retinal diseases, accumulating evidence points to the involvement of TMEM135 in the molecular pathways underlying such diseases. As more information is collected on TMEM135, we will gain a better understanding of how aging contributes to disease processes, thus providing invaluable insight for the creation of novel therapies and identification of promising biomarkers for those individuals who may be affected by these conditions in the future.

## Author contributions

ML: Conceptualization, Visualization, Writing – original draft, Writing – review & editing. PG: Conceptualization, Visualization, Writing – original draft, Writing – review & editing. SI: Conceptualization, Visualization, Writing – original draft, Writing – review & editing. AI: Conceptualization, Funding acquisition, Supervision, Visualization, Writing – original draft, Writing – review & editing.
